# Chitins and Chitosans as Immunoadjuvants and Non-Allergenic Drug Carriers

**DOI:** 10.3390/md8020292

**Published:** 2010-02-21

**Authors:** Riccardo A. A. Muzzarelli

**Affiliations:** Emeritus Professor, University of Ancona, Ancona, Italy; E-Mail: Muzzarelli.raa@gmail.it; Tel./Fax: +39-071-36206; Mobile: 348-0379279; http://www.chitin.it/

**Keywords:** chitin, chitosan, chitinase, chitinase-like proteins, immunology

## Abstract

Due to the fact that some individuals are allergic to crustaceans, the presumed relationship between allergy and the presence of chitin in crustaceans has been investigated. *In vivo*, chitin is part of complex structures with other organic and inorganic compounds: in arthropods chitin is covalently linked to proteins and tanned by quinones, in fungi it is covalently linked to glucans, while in bacteria chitin is diversely combined according to Gram(+/−) classification. On the other hand, isolated, purified chitin is a plain polysaccharide that, at the nano level, presents itself as a highly associated structure, recently refined in terms of regularity, nature of bonds, crystallinity degree and unusual colloidal behavior. Chitins and modified chitins exert a number of beneficial actions, *i.e.*, (i) they stimulate macrophages by interacting with receptors on the macrophage surface that mediate the internalization of chitin particles to be degraded by lysozyme and *N*-acetyl-β-glucosaminidase (such as Nod-like, Toll-like, lectin, Dectin-1, leukotriene 134 and mannose receptors); (ii) the macrophages produce cytokines and other compounds that confer non-specific host resistance against bacterial and viral infections, and anti-tumor activity; (iii) chitin is a strong Th1 adjuvant that up-regulates Th1 immunity induced by heat-killed *Mycobacterium bovis*, while down- regulating Th2 immunity induced by mycobacterial protein; (iv) direct intranasal application of chitin microparticles into the lung was also able to significantly down-regulate allergic response to *Dermatophagoids pteronyssinus* and *Aspergillus fumigatus* in a murine model of allergy; (v) chitin microparticles had a beneficial effect in preventing and treating histopathologic changes in the airways of asthmatic mice; (vi) authors support the fact that chitin depresses the development of adaptive type 2 allergic responses. Since the expression of chitinases, chitrotriosidase and chitinase-like proteins is greatly amplified during many infections and diseases, the common feature of chitinase-like proteins and chitinase activity in all organisms appears to be the biochemical defense of the host. Unfortunately, conceptual and methodological errors are present in certain recent articles dealing with chitin and allergy, *i.e.*, (1) omitted consideration of mammalian chitinase and/or chitotriosidase secretion, accompanied by inactive chitinase-like proteins, as an ancestral defensive means against invasion, capable to prevent the insurgence of allergy; (2) omitted consideration of the fact that the mammalian organism recognizes more promptly the secreted water soluble chitinase produced by a pathogen, rather than the insoluble and well protected chitin within the pathogen itself; (3) superficial and incomplete reports and investigations on chitin as an allergen, without mentioning the potent allergen from crustacean flesh, tropomyosine; (4) limited perception of the importance of the chemical/biochemical characteristics of the isolated chitin or chitosan for the replication of experiments and optimization of results; and (5) lack of interdisciplinarity. There is quite a large body of knowledge today on the use of chitosans as biomaterials, and more specifically as drug carriers for a variety of applications: the delivery routes being the same as those adopted for the immunological studies. Said articles, that devote attention to the safety and biocompatibility aspects, never reported intolerance or allergy in individuals and animals, even when the quantities of chitosan used in single experiments were quite large. Therefore, it is concluded that crab, shrimp, prawn and lobster chitins, as well as chitosans of all grades, once purified, should not be considered as “crustacean derivatives”, because the isolation procedures have removed proteins, fats and other contaminants to such an extent as to allow them to be classified as chemicals regardless of their origin.

## 1. Introduction

### 1.1. Recognition, allergy and asthma

Immunity against microbial pathogens primarily depends on the recognition of pathogen components by innate receptors expressed on immune and non-immune cells. Innate receptors are evolutionarily conserved proteins that recognize pathogens or products released by pathogens in different cellular compartments, such as the plasma membrane, the endosomes or the cytoplasm, and induce the expression of cytokines, chemokines and co-stimulatory compounds to eliminate pathogens and impart pathogen-specific adaptive immune responses.

Acute allergic sensitization in individuals involves Th2 cell expansion following exposure to allergens. Th2 cells secrete a multitude of cytokines, including IL-4, IL-5, IL-9 and IL-13, as well as chemokines such as thymus and activation-regulated chemokine and macrophage-derived chemokine, leading to further Th2 cell recruitment and activation of B cells. Under the influence of IL-4, B cells produce allergen-specific IgE that circulates and binds surface receptors on mast cells and basophils. Further exposure to allergen results in crosslinking of IgE on mast cells and basophils causing cell degranulation, releasing histamine, proteases, chemokines, prostoglandins, leukotrienes and a host of other mediators. This results in bronchoconstriction and recruitment of activated eosinophils, neutrophils, lymphocytes and macrophages. In some individuals, chronic allergic reactivity manifests as asthma due to airway wall remodeling, airways hyperresponsiveness and chronic inflammation. The allergic immune response was reviewed by Larche [1,2].

### 1.2. Chemistry of chitin associations in vivo and chitin isolates

There are several chitins of marine origin, but those commonly available from fishing activities are from crustaceans and squids. As described by Muzzarelli, they substantially differ from each other in terms of crystallinity; the crustacean chitins having the alpha polymorphic form, while the squid chitins exhibit the beta form that confers more versatility in chemical derivatization [3,4].

The present review article deals with crustacean chitins only, owing to the fact that the title matter is directly related to crustaceans. Because some individuals are allergic to crustaceans, some scientists have been prompted to investigate the presumed relationship between allergy and the presence of chitin in the crustaceans. For the first time in the scientific history of chitin, the well-assessed safety of chitin for the human organism has been questioned with unusually dramatic titles and figures of several articles. The problem has been set forth on a vague scientific basis, but with potential negative consequences on the exploitation of the chitin resources that derive from the industrial production of canned or frozen crustaceans.

While today, purified chitin itself can be used for some applications, in general its derivatives chitosan (obtained after deacetylation), chitooligomers (after partial depolymerization) and glucosamine with N-acetylglucosamine (after full depolymerization) are used in the biomedical field. These medical grade chemicals are further modified in many ways in order to optimize their performances. Indeed, they are refined carbohydrates with clearly described characteristic properties, and it would be unreasonable to see them as crustacean derivatives, even though their origin should be traceable. Even isolated chitin is a substance totally different from the chitin present *in vivo*, that is part of a complex structure with other organic and inorganic compounds. No instrumental analytical technique can identify the raw material from which a given sample of isolated chitin has been produced.

This is not a peculiarity of chitin, in any case. For example, maize starch commonly used for human consumption, is not regarded as a part of the maize plant: the extraction process of maize starch from the kernel is a process known as wet milling in which the starch is separated from the fiber, oil and protein. Similarly, with the aid of aggressive chemicals in a series of processes, chitin is separated from calcium carbonate, proteins and carotenoids. Both starch and chitin are the refined/purified polysaccharides with no more relationship with the respective living organisms that biosynthesized them.

Therefore, at the nano level, chitin is a highly associated structure that has been recently refined in terms of regularity, nature of bonds, crystallinity degree and other parameters. Certain laboratories have provided most valid and realistic description of the status of chitin in the crustaceans [5–7]. Two book chapters by Muzzarelli are useful for the comprehension of the structure of the arthropod cuticle and provide the essentials for the correct understanding of chitin and chitinases [8,9].

## 2. The Beneficial Activity of Chitin as an Immunoadjuvant

Nearly three decades ago, Suzuki *et al*. and Nishimura *et al*. demonstrated that chitin and chitin derivatives stimulate macrophages to produce cytokines that confer anti-tumor activity and nonspecific host resistance against bacterial and viral infections [10–14]. Since then, more specific immunologic activities of chitin have been reported. Shibata *et al*. re-evaluated the immunological effects of chitin *in vivo* and *in vitro* using phagocytosable small-sized chitin particles that demonstrated significant priming effects of chitin particles in alveolar macrophages and NK cells in mice: intravenous administration of fractionated chitin particles (1 to 10 μm) into the lung activates alveolar macrophages to express cytokines such as IL-12, tumor necrosis factor-α (TNFα), and IL-18, leading to INF-γ production mainly by NK cells [15]. Further studies by the same authors demonstrated that the cytokine production was through phagocytosis mediated by a mannose receptor [16]. The macrophage plasma membrane mannose receptors serve to mediate the internalization of the chitin particles that then are degraded by lysozyme and *N*-acetyl-β-glucosaminidase in the macrophages of human and experimental animals according to Bourbouze *et al*. [17]. The immunological aspects of chitin and chitin derivatives administered to animals were studied by Tokura *et al*. [18]. The studies cited here were the first demonstration of the direct interactions between chitin and cell surface receptors and indicated that chitin uses specific signaling pathways in immune regulation. Lee further demonstrated that chitin stimulates macrophages by interacting with different cell surface receptors such as macrophage mannose receptor, toll-like receptor-2 (TLR-2), C-type lectin receptor Dectin-1, and leukotriene 134 receptor (BLT1) [19]. The advances in the study of pathogen recognition and their signaling pathways have been discussed most recently [20–22].

The ample evidence that chitin is a potent innate immune stimulator of macrophages and other innate immune cells raises the possibility that chitin could affect allergen-induced adaptive type 2 responses as well, but, generally, type 1 cytokines are produced by innate immune cells and it has been shown that type I cytokines down-regulate type 2 allergic immune responses [23]. In addition, the administration of IFN-γ or IL-12 significantly inhibited Th2 driven inflammatory responses in allergic animal models [24,25]. Thus, it is reasonable that chitin depresses allergen-induced type 2 inflammatory responses. Several studies strongly support this approach. Shibata *et al*. have demonstrated that orally given chitin down- regulates allergen-induced IgE production and lung inflammation in a ragweed-immunized allergic animal model [23]. The allergen-stimulated production of Th2 cytokines, such as IL-4, IL-5, and IL-10 was inhibited by the presence of chitin in spleen cell culture. The IFN-γ produced by NK cells and Th1 cells was responsible for the inhibition of allergeninduced Th2 cytokine production. They have also shown that chitin is a strong Th1 adjuvant that up-regulates Th1 immunity induced by heat-killed *Mycobacterium bovis*, while down regulating Th2 immunity induced by mycobacterial protein [26]. The Th1 adjuvant effect of chitin microparticles in inducing viral specific immunity has also been reported by Hamajima *et al*. [27].

Strong *et al*. showed that direct intranasal application of chitin microparticles into the lung also significantly down-regulated allergic response to *Dermatophagoids pteronyssinus* and *Aspergillus fumigatus* in a murine model of allergy [28]. The chitin treatment substantially reduced the allergen-induced serum IgE levels, peripheral eosinophilia, airway hyper-responsiveness, and lung inflammation. They noted the elevation of Th1 cytokines IL-12, IFN-γ and TNF-α and reduction in IL-4 production in the chitin-treated mice compared to sham controls. Similarly, intranasal application of water soluble chitosan attenuated mucus production and lung inflammation induced by *Dermatophagoids farinae.*

Ozdemir *et al*. further demonstrated that application of microgram quantities of chitin microparticles had a beneficial effect in preventing and treating histopathologic changes in the airways of asthmatic mice [29]. All these studies strongly support the fact that chitin depresses the development of adaptive type 2 allergic responses. As a regulatory mechanism, down regulation of allergen-induced arginase I and thymic stromal lymphopoietin expression in the bronchial epithelium was suggested and discussed [30–32]. From the clinical point of view, the regulatory function of chitin on Th2 adaptive immune response is therapeutically important because it can be applied to control a variety of type 2 allergic diseases. These data indicate that the administration of chitin is beneficial (instead of dangerous) because it exerts immunoadjuvant effect, and depresses the insurgence of Th2 allergy. Shrimp chitin is therefore a potent T and B cell adjuvant when delivered in the form of chitin microparticles and can shift a polarized T-helper type 2 (Th2) immune response [allergy] towards a Th1 response [inflammation].

Advances made in the last quinquennium in the way of thinking about the chitin/chitinase/chitin-like proteins subject, can be illustrated by the contributions made by the Elias team, through a number of articles. In an introductory note to the article by Zhu *et al*. on the acidic mammalian chitinase in asthmatic Th2 inflammation [33], Couzin recommended caution in the interpretation of the available data “because something is missing” [34]. Then, the concept that asthma might be a parasite-independent antiparasite response was introduced by Elias *et al*. [35]; they compared the clinical data on chitinase 3-like-1 levels of certain groups of patients with severe asthma [36], and reminded that the chitinase 3-like-1 (otherwise called human cartilage glycoprotein-39, HCgp-39, or YKL-40) is also present in patients with meningitis, pneumonia, rheumatoid arthritis, osteoarthritis, breast and lung cancer, and hepatic fibrosis. Incidentally, abundant literature is available on the presence of the chitinolytic enzyme *N*-acetylglucosaminidase in patients suffering from most various diseases or particular physiological situations in which chitin is certainly not involved (for a review see [37]). They also underlined that antigens that contain chitin (dust mite *D. pteronyssinus*) and antigens that do not contain chitin (rye and birch) induce similar responses [38].

Lee *et al*. and DaSilva *et al*. reviewed the beneficial immunostimulating activity of chitin, and demonstrated that the chitin activity depends on the size of its particles (higher when particles are 2–10 micron) [39,40]. Most recently they reviewed the field of chitinase-like proteins whose main value resides in being novel biomarkers in asthma [41].

## 3. Chitinases and Chitinase-Like Proteins in Mammals

Mammals produce chitinases, and their increased secretion is closely associated with pathophysiological conditions dominated by T-helper type 2 cells (Th2) including infection, fibrosis, allergy and asthma [33,42,43]. The review by Sutherland *et al*. gives a broad perspective of the effects and functions of chitinases and CLPs in the context of their association with allergy and asthma [44]. Studies on chitinases in lower organisms may provide some interesting parallels for understanding the function of the mammalian chitinases and CLPs. The action and pattern of chitinase expression in plants, bacteria, viruses and fungi illustrate a number of diverse roles in morphogenesis, nutrition, defense and stress [45].

The most common feature of chitinase activity in all organisms appears to be the biochemical host defense against chitin-containing pathogens. In fact, in mammals, expression of chitinases and CLPs is greatly amplified during many infections [46]. Indeed, the chitinase-like protein, Ym1, was first reported as a prominent novel product in mice infected with the helminths *Trichinella spiralis, Brugia malayi* and *Schistosoma mansoni* [47–49].

The Ym1 production is strikingly associated with a distinct cell phenotype termed the alternatively activated macrophage (AAM). High levels of IL-4 and IL-13 in Th2-driven inflammatory settings of infection and allergy stimulate abundant numbers of AAMs [50] which also up-regulate resistin-like molecule-a, arginase-1 and the mannose receptor [51]. Hence, while fungal infections with *Cryptococcus neoformans* are normally limited by a Th1 response, in IFN-γ-deficient and IL-13 overexpressing mice there is a dominant Th2 response accompanied by alternative activation of macrophages and production of Ym1, associated with more severe disease [52, 53].

The abundant secretion of Ym1 by murine AAMs and alveolar macrophages suggests that it is required in some quantity to fulfil an as yet undefined role in inflammation and repair because Ym1 binds components of the extracellular matrix. One might speculate that chitinases and CLPs, through interactions with the extracellular matrix and host sugars, provide a physical basis for tissue repair and remodeling, appropriately during helminth infection and inappropriately during asthma.

Chitinases and CLPs are certainly prominent in the human response to infection, and human alveolar macrophages from allergic or asthmatic patients express the acidic mammalian chitinase, AMCase, which is also seen in mouse models of helminth infection and allergy. Elevated chitinase activity and/or protein levels in humans implicate AMCase, chitrotriosidase and chitinase 3-like-1 in severe infections. Human plasma levels of chitrotriosidase activity increase upon infection with fungal pathogens and malaria parasites, consistent with an involvement in host defense [54–56].

The roles of chitinases and CLPs in human host defense thus remain unresolved. From current knowledge it would appear that both chitinases and CLPs are strongly associated with both innate and adaptive immune responses, but direct evidence is still lacking that they play an effector role in anti-parasite immunity. Whether their primary function is in the context of protective immunity, or tissue repair, in different contexts these proteins may be either beneficial or detrimental for the host.

## 4. Misleading Statements and Sources of Errors

Chitin has been directly associated to asthma in a couple of articles on a rather arbitrary basis by Burton & Zaccone and by Dickey [57, 58]. The close examination of these articles indicates that the authors equivocated about the chemical form of chitins occurring *in vivo*. It is apparent that a number of authors are not familiar with books and articles describing the structure and the chemical/biochemical combinations of chitin *in vivo*, and they arbitrarily assumed that chitin in mites, worms, fungi and bacteria is something similar to the packaged chitin supplied by industrial producers. Basic books [59–67] do not appear to have been cited in their articles.

The animal/human organism has an alert system that detects pathogens, but it is highly questionable that isolated chitin itself could trigger and sustain a series of biochemical reactions leading to allergy and asthma particularly when said chitin is a part of a biological structure. In fungi, chitin is covalently linked to glucans, while in arthropods it is covalently linked to proteins and tanned by quinones; in bacteria chitin is diversely combined according to Gram(+/−) classification.

Therefore, illustrations and text in the article by Burton & Zaccone [57] are misleading because they translate the images of arthropods, fungi and helminths into the chitin chemical formula; it is stated there that “exposure to chitin from dust mites, moulds, shellfish and insects might be the primary external determinant in allergy development” without providing experimental proof. Similarly, Figure 1 and the corresponding legend in the article by Dickey [58] interpret the images of mites, fungi and helminths as chitin whose formula is placed in the middle of an “alveolus” in such a way as to give the impression that chitin is responsible for allergic inflammation in the airways. Rudimentary and approximate is the analogous Figure 1 in the review by Shuhui *et al*. [68]. Remarkably, all these authors forget to add bacteria to the list.

As said above, purified chitin is certainly an immunoadjuvant, but the aspect that escaped the observation of many research workers is the mechanism by which chitin-bearing pathogens are recognized by the mammalian organism: it is a well assessed fact that bacteria, mites, fungi and nematodes are recognized. Hong *et al*. [69] investigated the direct effects of exogenous chitinase from *Streptomyces griseus* on Ca^2+^ signaling in human airway epithelial cells: the exogenous chitinase was found to cleave a model peptide representing the cleavage site of protease-activated receptor-2 (PAR-2) and enhanced IL-8 production. These results indicate that exogenous chitinase is a potent proteolytic activator of PAR-2 that can directly induce Ca^2+^ signaling in human airway epithelial cells. The meaning of this is that the mammalian organism recognizes the secreted water soluble chitinase produced by a pathogen, rather than the insoluble and well protected chitin within the same pathogen. This is followed by the secretion of the mammalian chitinase and/or chitotriosidase, as a generic defensive means against invasion. According to Chen *et al*. direct inhibition of hyphal growth could be achieved with the use of AMCase; in fact, they studied the *in vitro* antifungal activity of acidic mammalian chitinase against *Candida albicans, Aspergillus fumigatus* and *Trichophyton rubrum* strains. The growth of all three dermato-pathogenic fungi was clearly inhibited by recombinant and natural AMCase, especially the natural one [70].

For example, the fungal pathogen *Candida albicans* has a multilayered cell wall composed of an outer layer of proteins glycosylated with *N*- or *O*-linked mannosyl residues and an inner skeletal layer of β-glucans combined with chitin. It was demonstrated that cytokine production by human mononuclear cells or murine macrophages was markedly reduced when stimulated by *C. albicans* mutants defective in mannosylation. Recognition of mannosyl residues was mediated by mannose receptor binding to *N*-linked mannosyl residues, and by TLR4 binding to O-linked mannosyl residues. Residual cytokine production was mediated by recognition of β-glucan by the dectin-1/TLR2 receptor complex. Recognition of *C. albicans* by monocytes/macrophages is mediated by three recognition systems of differing importance, each sensing specific layers of the C. albicans cell wall, but not chitin [71].

It was found that dectin-1 recognizes *C. albicans* yeast, but not filaments [72]. Deformities produced in the cell wall during budding expose patches of β-glucan that are recognized by the receptor. In fact, these patches, identified as bud and birth scars, are sufficient to permit activation of dectin-1, and trigger potent antifungal inflammatory responses in macrophages. *C. albicans* filaments do not expose β-glucan in bud scars since mother–daughter cell separation does not occur, and thus filamentous *C. albicans* avoids to activate dectin-1-mediated mammalian defenses. Therefore the *C. albicans* shape alone (not the presence of glucan, even less chitin) contributes to the approach by which phagocytes recognize the fungus.

In humans, distinct membrane Toll-like receptors can directly bind common bacterial components such as lipopolysaccharides, bacterial lipoprotein, and peptidoglycan and subsequently initiate an intracellular signaling pathway leading to innate immune gene induction. In *Drosophila*, the binding protein DGNBP-1 has high affinity to microbial immune elicitors such as lipopolysaccharide and β-1,3-glucan whereas no binding affinity is detected with peptidoglycan, β-1,4-glucan, or chitin. Importantly, the overexpression of DGNBP-1 in *Drosophila* enhances lipopolysaccharide- and β-1,3-glucan-induced innate immune gene expression. These results suggest that DGNBP-1 functions as a pattern recognition receptor for LPS from Gram(−) bacteria and β-1,3-glucan from fungi and plays an important role in non-self recognition and the subsequent immune signal transmission for the induction of antimicrobial peptide genes in the *Drosophila* innate immune system. Therefore, many organisms from humans to insects recognize bacteria without reference to chitin [73].

The name chitinase has been abused in some articles as well. The most recent articles correctly underline the fact that the compounds under study are deprived of enzymatic activity, *i.e.*, they are chitinase-like proteins, but besides the most often mentioned acidic mammalian chitinase (AMCase), other enzymes are not mentioned at all, such as *N*-acetylglucosaminidase, NAGase, which has been the object of studies for many years and still appears frequently in the literature. Therefore, even in this area, the ongoing studies ignore a large part of the enzymological knowledge gained in this field. The enormous body of information on NAGase includes its use as a biomarker of stress and renal functionality in patients, and its significance in key aspects of ecology such as parasitism. More specifically, NAGase is present in the epidermis of crustaceans (under hormonal control) where it is responsible for the resorption of chitin in the early stage of the molt. Nevertheless, those authors who claimed the involvement of chitin in asthma insurgence in workers exposed to crustaceans, did not care about the possible presence of NAGase in those hypothetically inhaled aerosols where they imply that insoluble chitin particles are present (not demonstrated). Even worse, they did not pay attention to the real allergens, tropomyosin *in primis*, a soluble compound coming from crustacean flesh. The superficiality and incompleteness of these investigations is such that not even a word was spent on NAGase and on tropomyosin. This methodological error remained undetected for a long time, and the arbitrary equivalence chitin = allergen was taken for granted [74].

The claim that fish factory workers may get asthma as a consequence of the exposure to “chitin” should be rejected. Based on what was said above, the claim is hardly sustainable in the light of the food technology actuated in shrimp and lobster handling: for example, the shrimps (either to be canned or to be frozen) are handled by peeling machines with minimal supervision by workers, with minimal fragmentation of the shells and with no aerosol dispersion. In those factories, protection of the workers and product quality assurance are mainly concerned with the effects of proteases and recognized allergens.

The major shrimp allergen has been identified as the muscle protein tropomyosin, which is also present in other crustaceans (lobsters, prawns and crabs) and a variety of marine organisms, but also in insects such as the cockroach and in arachnids such as house dust mites. In certain individuals this protein processed by macrophages and presented to T and B lymphocytes stimulates the production of protein-specific IgE antibodies with consequent insurgence of the allergy symptoms. The tropomyosins of various sources are listed in one of the tables in the article by Lehrer *et al*. [75].

Shrimp-derived glucosamine is safe even for individuals hypersensitive to tropomyosin. Villacis *et al*. state that glucosamine supplements from various manufacturers do not contain clinically relevant levels of allergens [76]. Gray *et al*. clearly state that “shellfish allergy is caused by IgE antibodies to antigens in the flesh of the shellfish and not the shell; therefore it should be safe for patients with shellfish allergy to take glucosamine supplements” [77].

Another error resides in the belief that any isolated chitin is suitable for research in the medical field. Reese T.A. *et al*. remarked that transgenic mice overexpressing AMCase showed no sign of spontaneous inflammation, whilst in a previous report of theirs AMCase was interpreted as a pro-inflammatory mediator. They expressed the view that AMCase has a role in the feedback attenuation of innate immune response against chitin-bearing pathogens, the said response being the enzymatic degradation of the pathogen’s chitin, thus removing the stimulus for further eosinophil and basophil recruitment [78]. The authors administered a chitin suspension to mice intraperitoneally or nasally: they wrote that the chitin (catalog code omitted) was supplied by New England Biolabs, but they did not mention origin, physico-chemical characteristics, and chemical purity; they simply declared that it was washed in phosphate buffer saline, but they did not specify unambiguously the final concentration of chitin in suspension. Surprisingly, the referees did not remark on such a flaw that prevents replicating the experiment. The claim that unknown quantities of isolated chitin of unknown purity induce accumulation in mice tissue of innate immune cells associated with allergy [78] might be unrealistic when referred to the actual quantities of chitin released by parasitic worms during moulting.

The controversy is also based on the already cited studies [15, 16, 23, 28, 29] that demonstrated that chitin was anti-allergenic, reducing allergen-induced increases in BALF eosinophils and lymphocytes, IgE levels, Th2 cytokines IL-4 and IL-5, goblet cell hyperplasia and subepithelial fibrosis. The down-regulatory effects of chitin on the allergic response may be partially due to the induction of Th1 cytokines. Chitin may induce a Th1 response in allergic animals to antagonize an already primed Th2 response. It is thus possible that an increase in chitin due to chitinase inhibition does not result in adverse effects in individuals already allergic.

Another error was to disregard the study of chitotriosidase, which was the first to be described as a human chitinase, according to the abundant literature on this enzyme. As a matter of fact, chitotriosidase did not appear in the literature mentioned here until 2007 when Barone *et al*. published a review [79]. In late November 2008, Seibold *et al*. published an article [80] on chitotriosidase as the primary active chitinase in the human lung, modulated by genotype and smoking habit. They stated that: (1) = the chitinase activity in broncho-alveolar lavage fluids matched that of chitotriosidase but not that of AMCase; (2) = AMCase transcripts in macrophages were consistent with an isoform lacking enzymatic activity; (3) = the chitinase activity in lavage fluids tended to be lower than normal in asthmatic subjects, but was increased 7-fold in habitual smokers; and (4) = chitinase activity did not increase in subjects with asthma and in fact tends to be decreased. Indeed, such results invalidated most of the previous research results, insofar as the major mammalian chitinase, chitotriosidase, was disregarded by all previous research workers, who attributed enzymatic activity to AMCase, which on the contrary might be present as an isoform deprived of enzymatic activity. Moreover, the asthmatic patients had lower-than-normal chitinase, whilst smokers had seven-fold higher. Here the importance of cigarette smoking is at the same level as the diesel exhaust mentioned [81] and the toxicity of diisocyanates.

## 5. Novel Views from Experimental Evidence

While studying the inflamed colon in the dextran sulfate-induced colitis model, the Mizoguchi lab found upregulated gene expression of chitinase 3-like-1 in colonic epithelial cells and macrophages in the inflamed colon: they demonstrated that chitinase 3-like-1 exacerbates intestinal inflammation by facilitating bacterial adhesion and invasion into the intestinal mucosa and the development of acute colitis [82,83]. No chitin/chitosan was used in these investigations. Neutralization of CHI3L1 *in vivo* ameliorated the induced colitis. Nevertheless, it is unlikely that CHI3L1 recognizes bacterial cells via their chitin component, because the external surface of the bacterium is essentially anionic due to the presence of teichoic acids and phosphorylated compounds, and because chitin is protected by glycoproteins and glucans. Interestingly, a positive correlation between serum CHI3L1 and *C*-reactive protein levels (CRP being an inflammation marker) was previously reported in ulcerative colitis patients. Therefore, it was deduced that CHI3L1 is upregulated in acute and chronic colitis: in fact, endogenous CHI3L1 mRNA level was found to be upregulated after the stimulation with pro-inflammatory cytokines (TNFα, IL-1β and IL-6) in colonic epithelial cell lines. The authors were extremely cautious when mentioning chitin: for example they wrote that it would be possible that CHI3L1 binds to a complex of chitin with chitin-binding proteins that is formed on bacteria and this binding may subsequently enhance the adhesion and invasion by bacteria. The claim “through the interaction with chitin” [84] was abandoned here and in subsequent 2008 publications by the same team.

In realistic terms, certain proteins could bind to the bacterial surface without involving the chitin. As for AMCase, the neutralization of its enzymatic activity *in vivo* by administration of anti-AMCase anti-body or a chitinase inhibitor (allosamidin) significantly alleviated airway inflammation as well as airway hyper-responsiveness. That study suggests that AMCase can be targeted to control asthma and other forms of Th2-mediated inflammation. On the other hand, if chitin was so powerful to elicit inflammation, the authors would have used it instead of dextran sulfate [82].

Dysregulated host-microbial interactions play a pivotal role in the pathogenesis of inflammatory bowel disease, and there is interest in maintaining the normal bacterial flora. The impression is that the chitinase-like proteins (CHI3L1) interact unspecifically with compounds (other than chitin) occurring on the bacterial cell surface. The adhesion rate of CBP21-overexpressing non-pathogenic *E. coli* was increased by two-fold in the presence of CHI3L1, that is therefore involved in the enhancement of adhesion of CBP-expressing bacteria to colonic epithelial cells. Chitin binding protein-21 (CBP21) and its homologs may be required for the CHI3L1-mediated enhancement of bacterial adhesion to colonic cells through the conserved amino-acid residues [85]. This is a further indirect evidence that bacterial chitin is not directly accessible being part of a complex structure.

Soon afterwards, the Mizoguchi lab recognized that the oral administration of chitin ameliorates chronic colitis: thus, chitin *per se* can not produce inflammation in this context [86]: this conclusion is in full agreement with those of the other teams mentioned above who used chitin to attenuate allergic hypersensitivity to pathogens, for example by intranasal application of chitin. A considerable amount of data exists on the anti-inflammatory activity of chitosan in the lower intestine under similar conditions (not discussed here).

Concisely, the following aspects of the research activity so far examined should be underlined:

1. A = Lack of interdisciplinary coordination is evident. Specifically, polysaccharide chemistry, bacteriology, chitin enzymology, and other disciplines were not adequately involved in these studies.
2. B = A number of authors have re-directed their research at more finely documented targets, and have rectified in part their previous findings, or have attributed more importance to the diagnostic use of the data on chitinases.
3. C = the chitinase 3-like-1 has been the object of a review article by Coffman who has correctly described the countless circumstances in which this protein is expressed (indirectly pointing at the scarce significance that occasional exposure to chitin might have on its upregulation) [87]. Moreover, the capacity of this protein to interact with many compounds is in favor of further complex investigations.
4. D = some genetic aspects emerged in the most recent literature: Chatterjee *et al*. tested 16 allergens, some of which exempt from chitin, and carried out a genetic research on the polymorphism of the acidic mammalian chitinase gene, CHIA. They established, for the first time, a significant relationship of CHIA with atopic asthma and with total IgE levels in serum; they pointed out that *ex novo* research on this gene may clarify the reasons for asthma insurgence [88].

Zhang *et al*., not really involved with chitin/chitosan, studied a murine model of asthma (ovalbumin-sensitized mice): each animal was sensitized with 20 μg of chicken egg albumin plus 2 mg of aluminum hydroxide [81]. These authors had previously reported that a diesel exhaust particle extract induces a linear increase in new protein expression. The proteins are lungkine, a recently described chemokine, a family of chitinases including Ym1, Ym2, and acidic mammalian chitinase (AMCase), gob-5, a protein that mediates mucus secretion, and surfactant protein-D, a lectin capable of modulating inflammatory responses. They remained at undetectable levels or at very low levels in broncho-alveolar lavage fluid of normal mice, but were abundantly increased in airway inflammation. Low levels of oxidative stress-induced antioxidant and cytoprotective responses while higher levels of oxidative stress induced inflammation or cellular apoptosis. This article provides indirect but clear evidence that the presence of proteins belonging to the chitinase family among those elicited by ovalbumin in the airways does not mean that the inflammation (asthma) was generated by chitin/chitosan *i.e.*, the substrate that they recognize (actually absent in this experimental work), therefore chitin/chitosan have no capacity of inducing an inflammatory reaction so severe as to generate asthma. Moreover, because diesel exhaust particles are ubiquitous and everybody is exposed to their presence, it is unimaginable that occasional chitin powder inhalation promote detrimental effect more severe than those of diesel exhaust particles. In that article, the proteins mentioned result to be useful oxidative markers [81].

## 6. Current Research Trends

Li G.P. *et al*. used chitosan nanoparticles containing plasmid DNA encoding house dust mite allergen for oral vaccination of mice. The DNA was fully complexed into chitosan-DNA nanoparticles, suggesting a 100% encapsulation efficiency, a further advantage of chitosan being that it provides significant protection to the plasmid. The orally administered chitosan nanoparticles induced IFN-gamma in serum and prevented subsequent Th2 cell-regulated specific IgE responses [89].

Atopy, or atopic syndrome, is an allergic hypersensitivity subject to hereditary influences. The aberrant expression of chitinase 3-like-1 (CHI3L1) is involved in the pathogenesis of inflammatory and allergic diseases. The genetic contribution of CHI3L1 gene to atopy was investigated by Sohn *et al*. using an integrated population genetic and molecular analysis: the results indicated that the polymorphism in the CHI3L1 promoter region is associated with the risk of atopy [90].

Several authors sustained these views and concluded that more works using knockout mice, recombinant chitinases and siRNA technology are required to investigate a potential role of chitinases in the pathogenesis of asthma [68]. For asthma therapy, structural details of AMCase will help guide the future design of specific and potent AMCase inhibitors such as methylallosamidin [91].

In their most recent review on this topic, Seibold and Burchard conclude that “interaction between many researchers examining diverse aspects of chitin-chitinase biology is needed including human studies (chitinase expression, genetics and biochemistry) coupled with epidemiological studies of chitin exposure and mouse studies of chitinase biology” [92]. Of course, researchers should also be aware of the fact that chitins and chitosans represent a single aminated polysaccharide whose biological performances depend not only on the degree of acetylation, but also on the acetylation pattern, the degree of crystallinity, the average molecular weight and its polydispersity, among other parameters. For example, the susceptibility of chitins/chitosans to hydrolysis by lysozyme is high in a narrow range of acetylation values, optimum for certain acetylation patterns, but modest or nil otherwise.

## 7. Trends in Major Biomedical Fields

As discussed above, the most refined techniques are being used to throw light on the biochemical reactions that involve chitin in certain physiological microenvironments. Thus, chitin was administered to animals in a variety of ways, *i.e.*, nasally, orally, intraperitoneally, or directly to the eyes and to the lungs. Of course, in the area of controlled drug delivery, a remarkably large scientific production is based on the use of drug vehicles administered in the same ways: thus, relatively large amounts of chitosan accompany therapeutic doses of drugs.

In said publications, there is no mention of any adverse effect of chitosan in terms of allergy or severe inflammation, rather occasional appreciation of the immunostimulating action of chitosan can be found, particularly when the studies concern the targeted delivery to tumors and metastases. The following selected references concerning the delivery of a drug via various routes support this statement.

### Ocular delivery

In a review article, Paolicelli *et al*. provided the reader with a description of the advances made in the ocular delivery of bioactive compounds by means of chitosan-based nanosystems, resulting in innovative ocular nanomedicines with significant impact on clinical practice [93].

### Targeted delivery to tumors

Chitosan nanoparticles incorporating anticancer drugs such as doxorubicin are suitable for targeting the tumors and for reducing the systemic cytotoxicity of the drugs. Chitosan on its own inhibits growth of cancer cells and induces apoptosis of bladder tumor cells via caspase-3 activation. According to Tan *et al*. who reviewed the literature on this topic, chitosan is offering serious perspectives of advances in the encapsulation of this class of drugs leading to increased rates of tumor cell death or tumor size reduction [94].

### Nasal delivery

Intranasal administration of PEG-g-chitosan nanoparticles to rabbits enhanced the absorption of insulin by the nasal mucosa to a greater extent than a suspension of insulin-PEG-g-chitosan and control insulin solution. PEG-g-chitosan nanoparticles are promising vehicles for insulin transport through the nasal mucosa thanks to their mucoadhesion, cationicity, biodegradability and safety [81].

### Vaginal delivery

Perioli *et al*. prepared vaginal mucoadhesive tablets including the bioadhesive polymers chitosan and polyvinylpyrrolidone in 1:1 ratio. Hydration, mucoadhesion, metronidazole release and antibacterial activity were the most important characteristics of tablets together with suitable compactness and hardness [95]. Chitosan citrate favored penetration enhancement and peptidase inhibition [96].

### Oral administration

The most recent review on oral drug delivery with the aid of chitosan is the one by Werle *et al*. [97]. A book chapter on the oral administration of chitosan viewed as a dietary supplement and a food technology commodity was published [98]. Li F. *et al*. demonstrated that a sustainable increase of TGF-beta protein in mouse intestinal tissue can be induced after oral administration by gavage of a TGF-beta expressing DNA vector packed in chitosan nanoparticles [99]. Concomitantly, a significant amelioration of ovalbumin-induced food allergy symptoms was observed.

### Delivery to the colon

The polyelectrolyte complex formed by sodium cellulose sulfate and chitosan is a drug carrier for a colon-specific delivery, that can be activated by the colonic microflora: this promising system [100] is just an example among the many publications on this specific matter.

### Wound dressing

The most exhaustive and recent reviews have been published by Muzzarelli [101,102]. It is amply demonstrated that the massive use of chitin and chitosan in the form of freeze-dried materials, elaborated conduits, scaffolds, hydrogels and more, are useful for the healing of wounded soft tissue, bone, nerve and cartilage. In experimental and pre-clinical surgical trials, the use of chitin/chitosan and their derivatives has never led to allergies or other diseases.

### Gene therapy

At present, the most elaborated application of chitosan in the area of drug delivery is the delivery of genetic material for gene therapy [103,104]. The large number of recent publications testify that chitosan is exempt from undesirable properties.

## 8. Conclusions

This review shows that chitin and chitosan are endowed with immunoadjuvant capacities that were discovered nearly 30 years ago and were confirmed in subsequent years. It also shows that the current research relevant to chitin on asthma and allergy does not adequately take into consideration certain interdisciplinary aspects, so that misunderstandings are possible due to questionable assumptions, omission of essential documentation, and approximate characterization of materials. Sources of errors have been spotted in some of the most recent articles. It is presently unwise to interpret chitin as an allergenic substance, more clinical and genetic research being needed. Crab, shrimp, prawn and lobster chitins, as well as chitosans of all grades, once purified, should not be considered as “crustacean derivatives” because the isolation procedures have removed proteins, fats and other contaminants to such an extent as to permit to classify them as chemicals regardless of their origin.

## Acronyms

AAM: alternatively activated macrophage
AMCase: acidic mammalian chitinase
CBP: chitin binding protein
CHI3L1: chitinase 3-like-1
CLP: chitin-like protein
Ig: immunoglobulin
IL: interleukin
INF: interferon
LPS: lipopolysaccharide
NAGase: N-acetylglucosaminidase
PEG: poly(ethylene glicol)
TGF: transforming growth factor
Th: T helper
TLR: toll-like receptor
TNF: tumor necrosis factor
